# Exploring approaches to weighting estimates of facility readiness to provide health services used for estimating input-adjusted effective coverage: a case study using data from Tanzania

**DOI:** 10.1080/16549716.2023.2234750

**Published:** 2023-07-18

**Authors:** Ashley Sheffel, Emily Carter, Debora Niyeha, Khadija I. Yahya-Malima, Deogratius Malamsha, Shagihilu Shagihilu, Melinda K. Munos

**Affiliations:** a Department of International Health, Johns Hopkins Bloomberg School of Public Health, Baltimore, MD, USA; b Hellen Keller International, Dar Es Salaam, United Republic of Tanzania; c School of Nursing, Muhimbili University of Health and Allied Sciences (MUHAS), Dar Es Salaam, United Republic of Tanzania; d National Bureau of Statistics, Dar Es Salaam, United Republic of Tanzania

**Keywords:** Coverage, quality of care, household survey, health facility assessment, LMIC, linking methods

## Abstract

The ideal approach for calculating effective coverage of health services using ecological linking requires accounting for variability in facility readiness to provide health services and patient volume by incorporating adjustments for facility type into estimates of facility readiness and weighting facility readiness estimates by service-specific caseload. The aim of this study is to compare the ideal caseload-weighted facility readiness approach to two alternative approaches: (1) facility-weighted readiness and (2) observation-weighted readiness to assess the suitability of each as a proxy for caseload-weighted facility readiness. We utilised the 2014–2015 Tanzania Service Provision Assessment along with routine health information system data to calculate facility readiness estimates using the three approaches. We then conducted equivalence testing, using the caseload-weighted estimates as the ideal approach and comparing with the facility-weighted estimates and observation-weighted estimates to test for equivalence. Comparing the facility-weighted readiness estimates to the caseload-weighted readiness estimates, we found that 58% of the estimates met the requirements for equivalence. In addition, the facility-weighted readiness estimates consistently underestimated, by a small percentage, facility readiness as compared to the caseload-weighted readiness estimates. Comparing the observation-weighted readiness estimates to the caseload-weighted readiness estimates, we found that 64% of the estimates met the requirements for equivalence. We found that, in this setting, both facility-weighted readiness and observation-weighted readiness may be reasonable proxies for caseload-weighted readiness. However, in a setting with more variability in facility readiness or larger differences in facility readiness between low caseload and high caseload facilities, the observation-weighted approach would be a better option than the facility-weighted approach. While the methods compared showed equivalence, our results suggest that selecting the best method for weighting readiness estimates will require assessing data availability alongside knowledge of the country context.

## Introduction

In low- and middle-income countries (LMICs), effective coverage estimates of health service provision are often generated by linking data from household surveys and health facility assessments (HFAs) [1]. The ideal approach is exact-match linking whereby information for each individual seeking care in the household survey is linked to information about the quality of care of the specific health facility visited. However, this is generally not feasible for large-scale, national surveys in LMICs [2]. An alternative is ecological linking, whereby each individual seeking health care services in the household survey is linked to an average quality of care score of health facilities within the same administrative area as the household [3–5].

Health facilities within an administrative area are often of varying types (hospitals, health centres, health posts) and as such they are often not equally ready to deliver care and they do not care for equal volumes of patients. It is important to account for this variability when generating linked estimates as it reduces bias and results in a closer approximation to an exact-match link. This can be accomplished by incorporating adjustments for facility type into estimates of facility readiness, where readiness refers to the capability of health facilities to provide a service of minimum acceptable standards, and is measured by the availability of both physical resources and human resources, and by weighting facility readiness estimates by service-specific caseload [3–6].

Data on facility type is readily available in HFAs and incorporating adjustments for facility type into estimates of facility readiness is a recommended best practice for ecological linking. However, caseload data is not collected in standard HFA tools. It can also be difficult to obtain caseload data and link it to HFA data. The aim of this study is to use data from Tanzania to compare the ideal caseload-weighted facility readiness approach to two alternative approaches: (1) facility-weighted readiness and (2) HFA observation-weighted readiness, to assess the suitability of each approach as a proxy for caseload-weighted facility readiness.

## Methods

### Data sources

#### Tanzania Service Provision Assessment 2014–2015 (TSPA)

The 2014–2015 TSPA was a health facility assessment that included a standard set of survey instruments: a facility inventory questionnaire, health worker interviews, observation of ANC consultations, and exit interviews with ANC clients. The survey was sampled to be nationally representative with health facilities selected using stratified systematic probability sampling with stratification by region and facility type (equal probability within strata) with oversampling of hospitals (see Box 1 for details on facility types). The TSPA sampling frame was comprised of a master facility list (MFL) compiled by the Ministry of Health, Community Development, Gender, Elderly, and Children (MOHCDGEC) on mainland Tanzania. Strata were established by crossing region (25 mainland regions) and facility type (hospital, health centre, dispensary, and clinic). These strata were then used to select health facilities by stratified systematic probability sampling. In addition, hospitals were oversampled to include all hospitals in the country. The client sampling frame was comprised of the expected number of ANC clients present on the day of the survey as reported by the health facility. Clients were randomly selected for observation during their visit based on the expected number of ANC clients on the day of the visit. Observation of client–provider interactions was completed for a maximum of five clients per service provider, with a maximum of 15 observations in any given facility. The TSPA final report contains comprehensive information on the survey methodology and questionnaires [7]. The TSPA dataset is publicly available from the DHS Program data repository but has been de-identified to exclude health facility names.

**Box 1**: **Facility types in Tanzania [8]**

Facility types in Tanzania include the following:

- **Dispensary** – Dispensaries are generally the first point of contact with the health care system and are staffed by a clinical assistant, often with the support of a nurse. Services provided at dispensaries include maternal and child health care, assistance with uncomplicated deliveries, and basic outpatient curative care.
- **Health centre** – Health centres supervise the dispensaries and are staffed by a clinical officer often with the assistance of a clinical assistant, maternal and child health aide, health aide, and a health assistant. Services provided at health centres include preventative care as well as curative care for common diseases and minor surgery.
- **Clinic** – Clinics are private primary-level health facilities that provide mostly outpatient curative services. They employ nurses/midwives, clinical officers, doctors, and pharmaceutical technicians.
- **Hospital** – This includes national referral hospitals, regional hospitals, district hospitals, and private hospitals. Secondary care is provided by district hospitals, which are staffed by medical doctors and assistant medical officers supported by clinical officers and nurses. District hospitals offer both inpatient and outpatient services not available at lower-level facilities including laboratory, imaging, and surgical services. Tertiary care is provided at regional hospitals and national referral hospitals, which are staffed by surgeons and medical doctors, as well as general and specialised nurses and midwives. They offer similar services as district hospitals; however, they are larger, employ specialists in various fields, and offer additional advanced services.

#### TSPA Facility names dataset

The TSPA Facility names dataset contains the health facility names and unique IDs that can be linked to the 2014–2015 TSPA dataset. This data was made available by the Tanzania National Bureau of Statistics as part of the National Evaluation Platform (NEP) project implementation.

#### Tanzania health facility registry

The Health Facility Registry (HFR) is a web-based system used to provide public access to a database of information about all health facilities in mainland Tanzania. The HFR registry contains the name, region, and facility ID for each health facility and can be accessed at https://hfrportal.moh.go.tz/.

#### Tanzania DHIS-2 service utilisation data

Data on the number of ANC4+ visits (defined as a count of all ANC visits made that were the 4th or more visit for a woman) was extracted from the Tanzania DHIS-2 portal per month and per facility for 2015.

### Caseload estimates

The data on ANC4+ visits was exported from DHIS-2 for each region to a .csv file. All non-facility-level entries were dropped (i.e. district and region values), and duplicate entries were dropped. We then assessed the extent of missingness of ANC4+ data across the 12 months and created a binary variable with 0 indicating ≤5 months of missing data and 1 indicating >5 months of missing data. Facilities with more than five months of missing data were excluded from the analysis. For remaining facilities, missing data was imputed with the facility-specific mean value of ANC4+ visits (i.e. average monthly number of ANC visits across the months of available data for an individual facility). ANC4+ caseload was calculated as the total number of ANC4+ visits across the 12-month period (January–December 2015).

### ANC readiness score

We defined facility readiness as the human resources, equipment and supplies, diagnostics, medicines, and basic amenities essential to deliver a high-quality ANC service. A total of 19 items from the TSPA were selected to include in the facility readiness index. Details on item selection have been previously published [9]. For each item, a facility received one point if the requirements were met and a zero if not. A simple additive approach was utilised to calculate the index by taking the sum of the items available, dividing by the total number of items in the index, and multiplying by 100 to create a score between 0 and 100.

### Linking TSPA and DHIS-2 data

We used the HFR dataset to link the TSPA and DHIS-2 datasets. We merged the 2015 TSPA and DHIS-2 datasets using the HFR registry to identify facilities by both their name (TSPA) and facility identification code (DHIS-2).

### Calculate weighted facility readiness estimates

We aggregated estimates of facility quality over select categories of facility type, managing authority, and geographic unit aligning with different levels of aggregation that could be employed in ecological linking. Specifically, we generated aggregate quality scores by facility type, managing authority, urbanicity, and region. We limited the analysis to facilities that offered ANC services, had complete data for all facility readiness variables, and had at least 7 months of routine data available to calculate caseload. We weighted our aggregate facility readiness estimates using three approaches.

#### Approach 1: facility-weighted

The TSPA survey dataset includes survey weights (health facility weight, provider weight, client weight) that are calculated by the DHS Program. The health facility weight is calculated based on the health facility selection probability, adjusted for non-response at the sampling stratum level. The complex sampling design employed by the TSPA results in an unequal probability of facility selection. The use of sampling weights addresses the difference between the survey design and simple random sampling and ensures that the contribution of facilities to the total is proportionate to their existence in the country. Sampling weights do not account for facility use or caseload. The facility-weighted approach used only the TSPA health facility weight to generate the weighted facility readiness estimate, scaling each facility’s contribution to align with the national distribution of facilities.

#### Approach 2: observation-weighted

For the observation-weighted approach, we approximated differentials in client volume by multiplying each client’s weight by the number of client observations. The client weight is calculated by taking the health facility weight multiplied by the inverse selection probability of clients within each of the sampling strata, adjusted for client non-response. By scaling the client weight by the number of observations, the contribution of each facility’s readiness is adjusted to approximate the client load on the day of the survey. In addition, we limited the client observation dataset to women for whom facility-level data was included in the analysis (i.e. in mainland Tanzania, offered ANC services, complete data for all facility readiness variables, and at least 7 months of routine data).

#### Approach 3: caseload-weighted

For the caseload-weighted approach, we multiplied the health facility weight by the ANC4+ caseload estimate to generate a caseload weight. We applied these caseload weights when calculating mean facility readiness by category.

### Analysis

All estimates were generated using the R ‘survey’ package [10]. In addition to specifying the weights for each approach, we also accounted for the HFA sampling design, including cluster (health facility) and stratification (facility type and region). We then conducted equivalence testing, using the caseload-weighted estimates as the ideal approach and comparing with the facility-weighted estimates and observation-weighted estimates to test for equivalence with an equivalence interval of (−5% to 5%). Equivalence testing is an approach that tests for an effect that is large enough to be worthwhile examining. A widely recommended approach is to test for equivalence using the two one-sided tests (TOST) procedure, which specifies a lower and upper bound, such that results falling within this range are deemed equivalent to the absence of an effect that is worthwhile to examine [11]. The results of the TOST procedure present information on the classic null-hypothesis significance test (aka NHST) and the alternative equivalence hypothesis test (aka TOST). All statistical analyses were carried out using R version 4.1.3 [12].

## Results

### Survey characteristics

The 2014–2015 TSPA data set contained data from 1078 health facilities in mainland Tanzania, of which 949 provided ANC services. All 949 facilities offering ANC services had complete data for all facility readiness variables, while 860 facilities had at least 7 months of routine data available to calculate caseload. On average, each facility had an annual caseload of 45 ANC4+ clients. The annual health facility ANC4+ caseload ranged from 2 to 891. There was a total of 3466 ANC client consultations from 689 facilities observed in the 2014–2015 TSPA. On average, each facility had 5 ANC client observations. The number of ANC client observations at a health facility ranged from 1 to 15.

### Variability in caseload

**Figure 1.**
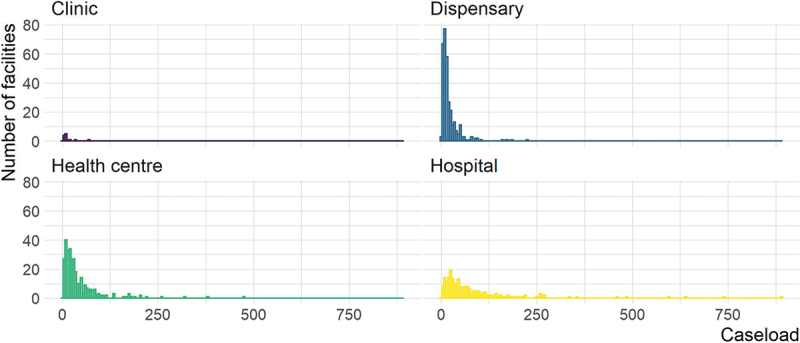
Histograms of caseload by facility type.

Caseload was variable both across and within facility type. Clinics and dispensaries tended to have lower caseload, while health centres and hospitals tended to have comparatively higher caseload. However, within health centres and hospitals, there were facilities with both very low caseload and very high caseload (Figure 1).

### Correlation between caseload and number of ANC observations

**Figure 2.**
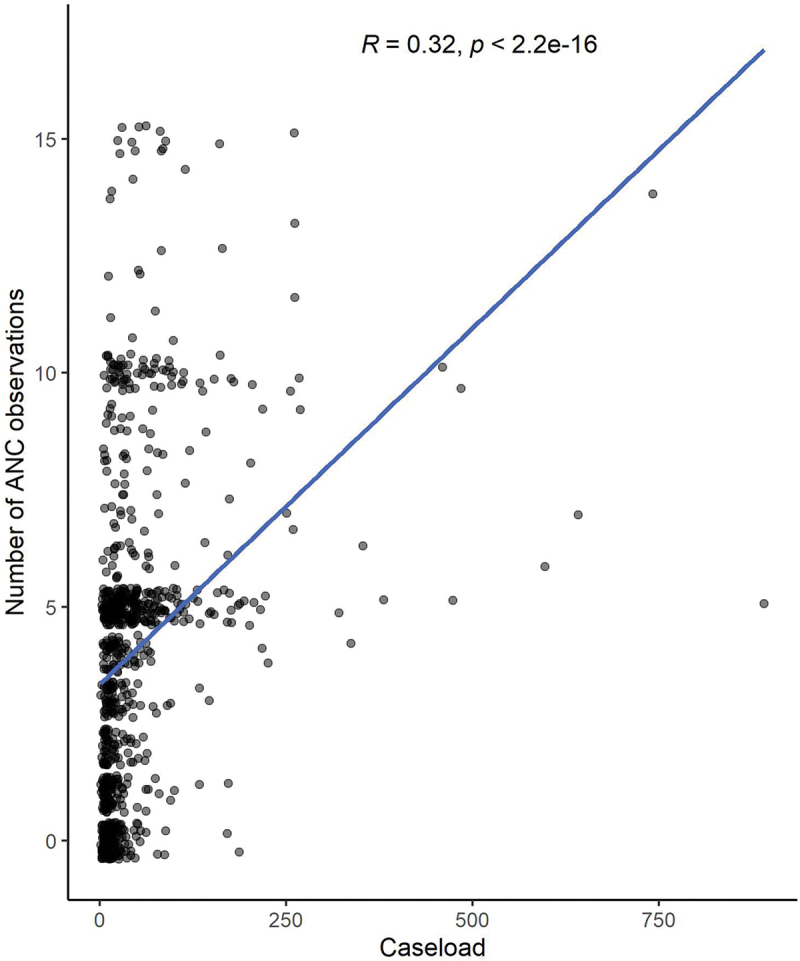
Correlation between caseload and number of ANC observations.

There was a weakly positive correlation (*R* = 0.32) between caseload and the number of ANC observations at a health facility (Figure 2). To ensure that this association was not driven by outliers, we examined the correlation among facilities with a caseload of 250 or less and found the correlation between caseload and the number of ANC observations at a health facility remained similar (*R* = 0.35).

### Facility readiness estimates and equivalence

The detailed results comparing the ideal caseload-weighted facility readiness approach to facility-weighted readiness and observation-weighted readiness are in Table 1. At the national level, comparing the facility-weighted readiness estimate to the caseload-weighted readiness estimate, we found that the estimates were statistically significantly different, but equivalent with a difference between caseload-weighted and facility-weighted estimates of 3.7%. Similarly, comparing the observation-weighted readiness estimate to the caseload-weighted readiness estimate, we found the estimates were statistically significantly different, but equivalent with a difference between caseload-weighted and observation-weighted estimates of −0.1%. This finding (statistically significantly different, but equivalent) demonstrates a reasonable approximation of the ideal caseload approach.

**Table 1.** Comparison of caseload-weighted readiness to facility-weighted readiness and observation-weighted readiness, by facility type, managing authority, urban/rural, and region.

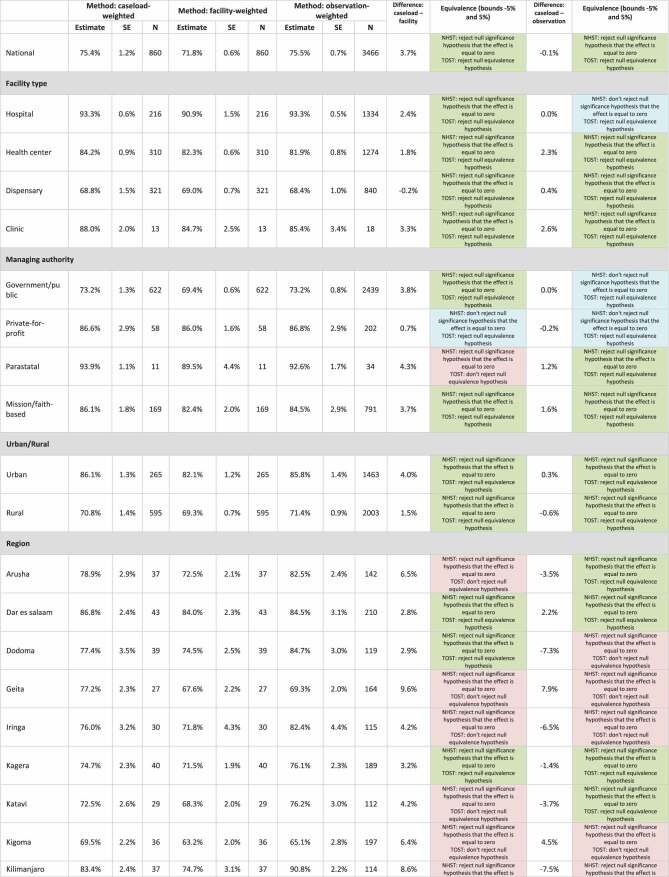
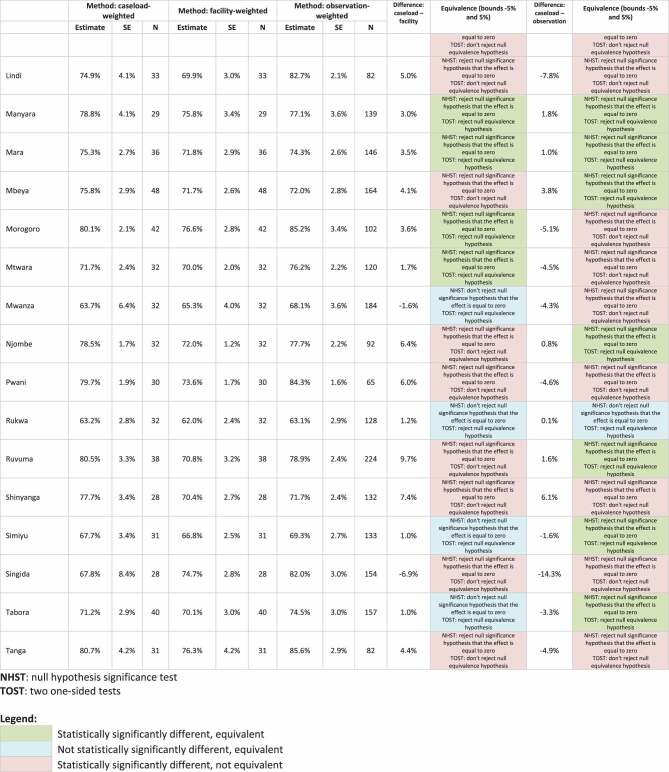

There was more variation when exploring results disaggregated by facility type, managing authority, urban/rural, and region. Comparing the facility-weighted readiness estimates to the caseload-weighted readiness estimates disaggregated by facility type, we found all facility-type estimates were statistically significantly different, but equivalent with a difference between caseload-weighted and facility-weighted estimates of −0.2% to 3.3%. Comparing the observation-weighted readiness estimates to the caseload-weighted readiness estimates, we found three out of four estimates were statistically significantly different, but equivalent with a difference between caseload-weighted and observation-weighted estimates of 0.4% to 2.6%. One estimate (hospitals) was not statistically significantly different and equivalent, which is what we would expect to see if the method was an exact approximation of the ideal caseload approach. Comparing the facility-weighted readiness estimates to the caseload-weighted readiness estimates disaggregated by managing authority, we found two out of four managing authority estimates were statistically significantly different, but equivalent with a difference between caseload-weighted and facility-weighted estimates of 3.7% to 3.8%. One estimate (private-for-profit) was not statistically significantly different and equivalent. One estimate (parastatal) was statistically significantly different and not equivalent with a difference between caseload-weighted and facility-weighted estimates of 4.3%. This finding (statistically significantly different and not equivalent) demonstrates this is not a reasonable approximation of the ideal caseload approach. Comparing the observation-weighted readiness estimates to the caseload-weighted readiness estimates, we found two out of four estimates were statistically significantly different, but equivalent with a difference between caseload-weighted and observation-weighted estimates of 1.2 to 1.6%. Two estimates (government/public and private-for-profit) were not statistically significantly different and equivalent. All urban/rural estimates comparing the facility-weighted readiness estimates to the caseload-weighted readiness estimates and the observation-weighted readiness estimates to the caseload-weighted readiness estimates were statistically significantly different, but equivalent with a difference between caseload-weighted and facility-weighted estimates of 1.5 to 4.0% and a difference between caseload-weighted and observation-weighted estimates of −0.6% to 0.3%. Comparing the facility-weighted readiness estimates to the caseload-weighted readiness estimates disaggregated by region, we found that 11 out of 25 regional estimates were statistically significantly different, but equivalent with a difference between caseload-weighted and facility-weighted estimates of 1.7% to 3.6%. Four estimates were not statistically significantly different and equivalent. Fourteen estimates were statistically significantly different and not equivalent with a difference between caseload-weighted and facility-weighted estimates of −6.9% to 9.7%. Comparing the observation-weighted readiness estimates to the caseload-weighted readiness estimates, we found 11 out of 25 regional estimates were statistically significantly different, but equivalent with a difference between caseload-weighted and observation-weighted estimates of −3.7% to 3.8%. One estimate was not statistically significantly different and equivalent. Thirteen estimates were statistically significantly different and not equivalent with a difference between caseload-weighted and facility-weighted estimates of −14.3% to 7.9%.

Looking across all estimates, comparing the facility-weighted readiness estimates to the caseload-weighted readiness estimates, we found 14% of the estimates were not statistically significantly different and were equivalent, which is what we would expect to see if the method was an exact approximation of the ideal caseload approach. An additional 44% of the estimates were statistically significantly different but were equivalent, which can be considered reasonable approximations of the ideal caseload approach. Combined, 58% of the estimates met the requirements for equivalence, while 42% of the estimates were statistically significantly different and not equivalent. The non-equivalent facility-weighted estimates were all regional estimates plus one managing authority estimate (parastatal). The facility-weighted readiness estimates consistently underestimated, by a small percentage, facility readiness as compared to the caseload-weighted readiness estimates with 33 out of 36 (92%) of the facility-weighted readiness estimates being lower than the caseload-weighted readiness estimates.

Looking across all estimates, comparing the observation-weighted readiness estimates to the caseload-weighted readiness estimates, 11% of the estimates were not statistically significantly different and were equivalent. An additional 53% of the estimates were statistically significantly different but were equivalent, for a total of 64% of the estimates meeting the requirements for equivalence. Thirty-six per cent of the estimates were statistically significantly different and not equivalent. The non-equivalent observation-weighted estimates were all regional estimates. There was no consistent trend in overestimation or underestimation when comparing the observation-weighted readiness estimates to the caseload-weighted readiness estimates.

## Discussion

We found that, in this setting, both facility-weighted readiness and observation-weighted readiness may be reasonable proxies for caseload-weighted readiness, as the national readiness estimates and readiness estimates disaggregated by facility level, urbanicity, and managing authority all met the criteria for equivalency (except for the parastatal estimate for facility-weighted readiness).

However, we found that the facility-weighted estimates consistently underestimated facility readiness, albeit by a small percentage, which occurred because facilities with higher readiness scores (which tended to have higher caseloads) were consistently upweighted in the caseload-weighted approach. In a setting with more variability in facility readiness or larger differences in facility readiness between low caseload and high caseload facilities, this underestimation could be more pronounced, and the observation-weighted approach would be a better option than the facility-weighted approach.

This study had a number of limitations. Data quality challenges with the routine data utilised for calculating caseload created an imperfect measure for our ‘ideal’ approach. We addressed some of the data quality issues by imputing missing data and excluding facilities with more than 40% missing data. Data quality is often a challenge for routinely collected data in LMICs, so this analysis likely reflects what is possible to achieve in other similar contexts [13,14]. This study was performed using data from a single country, which may limit generalisability. However, this approach could easily be replicated in other contexts with caseload data that could be linked to health facility data.

This study has provided an important contribution to the growing evidence around best practices for generating effective coverage estimates. While the methods compared showed equivalence, our results suggest that selecting the best method for weighting readiness estimates will require assessing data availability alongside knowledge of the country context.

## Paper context

Effective coverage is increasingly used to monitor universal health coverage, often by linking household surveys and health facility assessments. This paper provides a comparison of three methods for calculating weighted facility readiness for effective coverage and concludes that in Tanzania the three methods yield equivalent estimates. However, in a setting with more variability in readiness, facility-weighting may underestimate readiness. Insights from this study serve to advance best practices in methodologies for generating effective coverage estimates.
